# Totally endoscopic concomitant aortic and mitral valve surgery in junctional epidermolysis bullosa: a case report

**DOI:** 10.1186/s13019-024-02567-4

**Published:** 2024-02-20

**Authors:** Kazufumi Yoshida, Soshi Yoshida, Yoshimasa Hori, Hideki Tsubota, Ryosuke Mochizuki, Tohru Nagano, Tadaaki Koyama

**Affiliations:** 1 Department of Cardiovascular Surgery, Kobe City Medical Center General Hospital, 2-1-1 Minatojimaminamimachi, Chuo-ku, Kobe, 650-0047 Japan; 2 Department of Dermatology, Kobe City Medical Center General Hospital, Kobe, Japan

**Keywords:** Epidermolysis bullosa, Junctional epidermolysis bullosa, Minimal invasive cardiac surgery, Endoscopic, Aortic valve replacement, Sutureless valve, Mitral valve repair, Skin protection, Dressing foam

## Abstract

**Background:**

Junctional epidermolysis bullosa is a rare skin and mucosal disorder characterized by blister formation in response to minor trauma and extracutaneous manifestations. There have been no reports of cardiac surgery and prognostication in patients with epidermolysis bullosa due to skin and mucosal fragility.

**Case presentation:**

A 55-year-old man presented with congenital junctional epidermolysis bullosa, hypertension, and vasospastic angina. He complained of dyspnea on exertion, and transthoracic echocardiography revealed severe aortic valve regurgitation, moderate aortic valve stenosis (tricuspid valve), and severe mitral valve regurgitation. Considering that the skin condition in the right chest wall was relatively healthy, the right thoracotomy approach was preferred and totally endoscopic concomitant mitral valve repair and aortic valve replacement were performed using a sutureless bioprosthetic valve (Perceval™ (Corcym, Group, Milan, Italy)). Polyurethane and silicon dressing foams were used to protect the skin at the site of contact with the bag valve mask, arterial pressure catheter, intravenous catheter, and the tracheal intubation tube. Vertical mattress sutures were used for the skin sutures. The postoperative course was uneventful, and the patient was discharged nine days after the operation. There was no indication for reoperation until three years follow-up period.

**Conclusions:**

The totally endoscopic concomitant aortic and mitral valve surgery using Perceval™ prosthesis can be performed safely in patients with junctional epidermolysis bullosa by adequate protection of the skin and mucosa.

## Background

Epidermolysis bullosa (EB) is a rare congenital genetic condition that results in painful blistering of the skin and mucous membranes, which occurs with minor trauma or friction. The severity of EB can range from mild to fatal, and there is currently no cure for this condition [1]. Junctional epidermolysis bullosa (JEB) is a major form of epidermolysis bullosa. The milder form of junctional epidermolysis bullosa is called the JEB generalized intermediate and is typically associated with better prognosis [2]. Owing to the fragility of the skin in patients with EB, surgical procedures can be a challenge that requires special consideration. There have been no reports of cardiac surgery and prognostication in patients with EB owing to skin and mucosal fragility.

We report a case of a patient with JEB who underwent totally endoscopic concomitant mitral valve repair and aortic valve replacement. The present study also aimed to discuss the considerations of surgical techniques and perioperative management for JEB.

## Case presentation

**Fig. 1:**
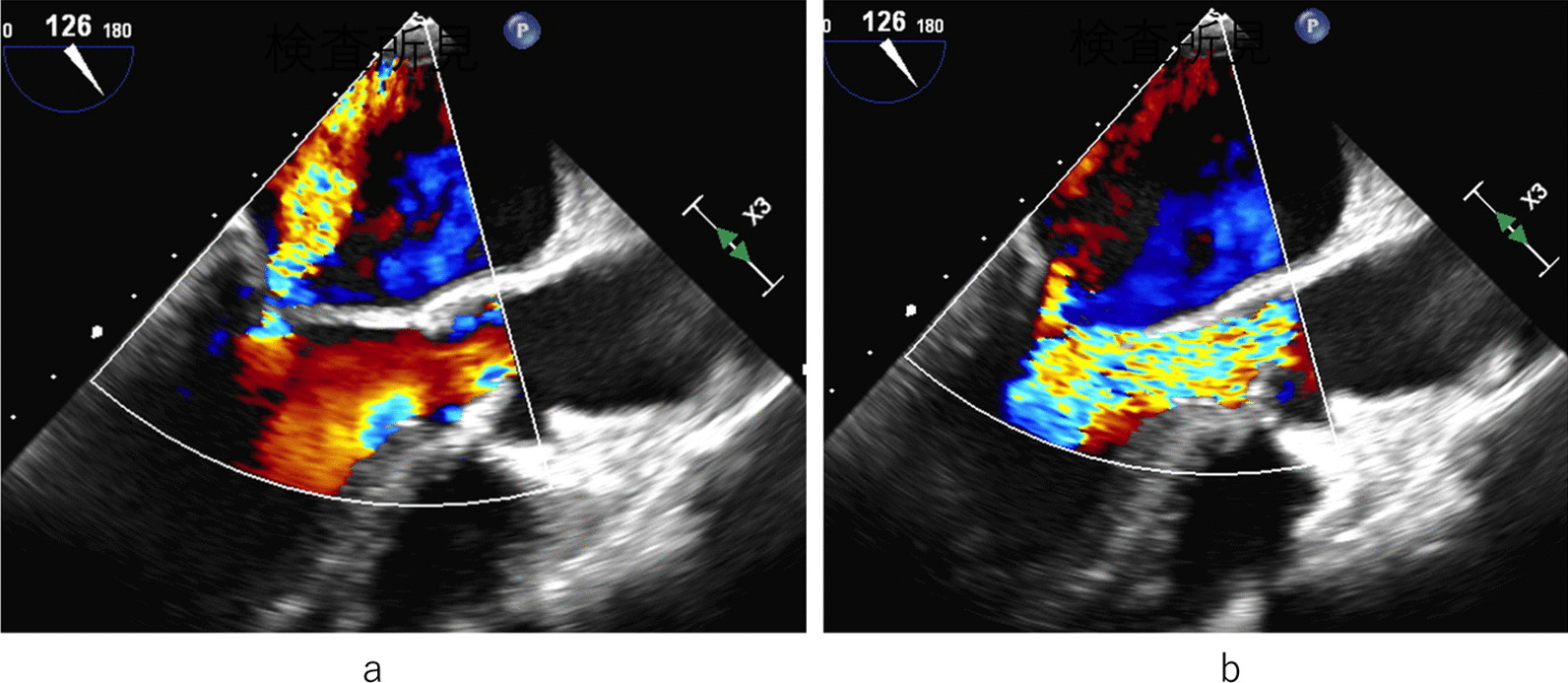
Preoperative transesophageal echocardiography. **a** Severe mitral valve regurgitation with dilated mitral annulus. **b** Thickening aortic valve (tricuspid) and severe aortic regurgitation

**Fig. 2:**
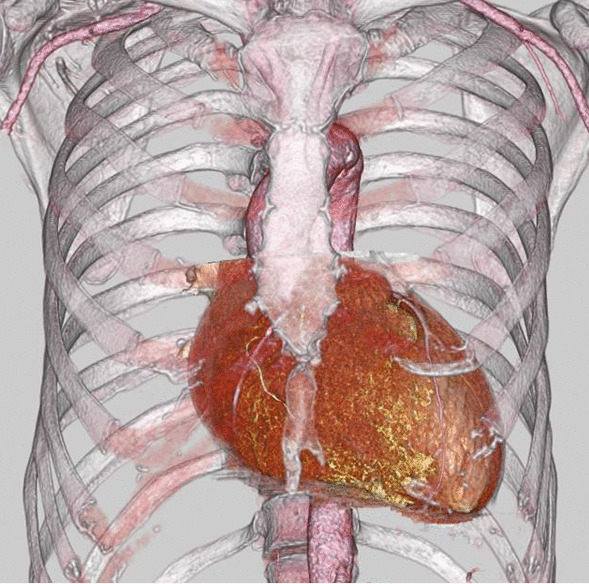
Preoperative computed tomography

**Fig. 3:**
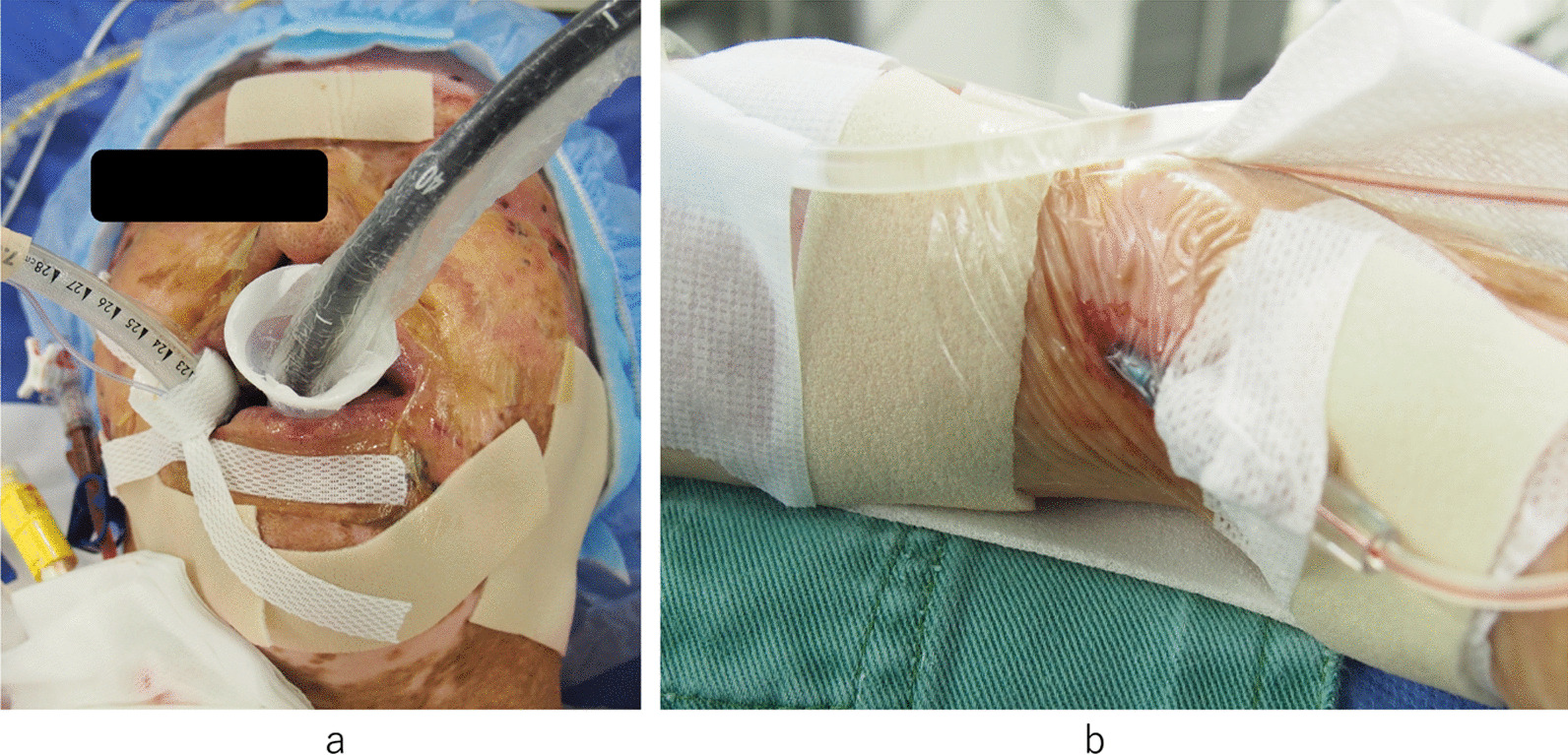
Skin protection with the polyurethane and silicon dressing foams. **a** Protections from the bag valve mask, intravenous catheter, and tracheal intubation tube. **b** Protection from arterial pressure catheter

**Fig. 4:**
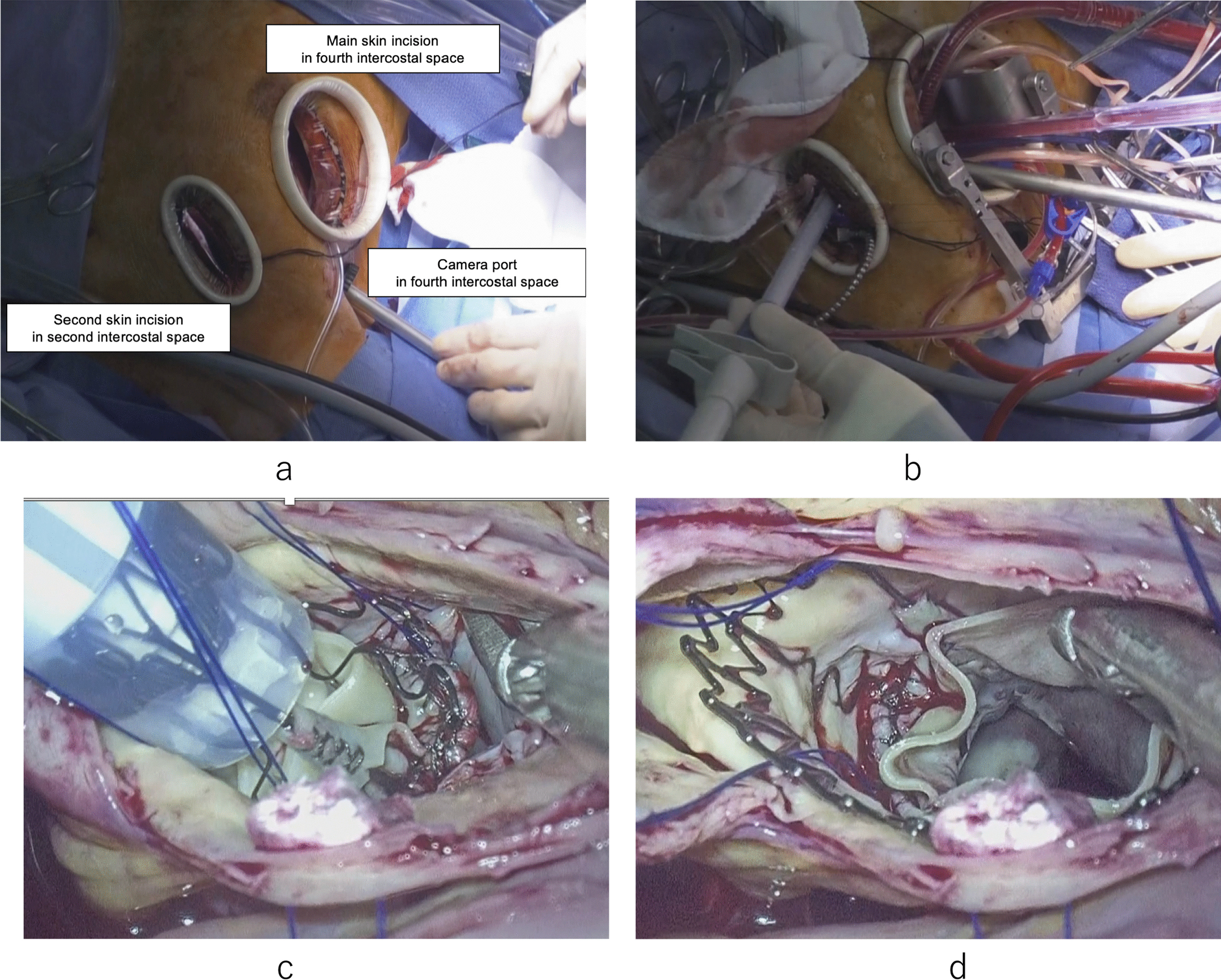
Totally endoscopic concomitant mitral and aortic valve surgery. **a** Three incision sites. **b** Insertion of Perceval prosthesis from the second incision site. **c** Before implantation of the prosthesis. **d** After implantation of the prosthesis

A 55-year-old male was a known case of JEB, hypertension, and vasospastic angina, who was treated with diltiazem hydrochloride and nicorandil. The patient underwent medical follow-up every 3 month at dermatology in other hospital. The chief complaint upon consultation was dyspnea on exertion. Chest radiography revealed a dilated cardiac shadow (cardiothoracic ratio, 59%), and electrocardiogram showed sinus rhythm within normal limits. Transthoracic echocardiography (TTE) revealed the following: left ventricular diastolic diameter (LVDd) / systolic diameter (LVDs) was 57/41 mm, left ventricular end-diastolic volume/end-systolic volume was 195.5/72.0 ml, left ventricular ejection fraction was 63.2% with no asynergy, severe aortic valve regurgitation (thickening tricuspid valve), peak velocity/mean pressure gradient of the aortic valve was 3.9 m/s/33.8 mmHg, severe mitral valve regurgitation with a dilated valvular annulus, and tricuspid regurgitation pressure gradient was 22.6 mmHg. Transesophageal echocardiography (TEE) revealed thickening of the aortic valve (tricuspid), severe aortic regurgitation, and severe mitral valve regurgitation (annulus dilatation) [Fig. 1]. The annulus, valsalva, and sinotubular junction sizes of the aorta were 21 mm, 30 mm, and 25 mm, respectively. Enhanced computed tomography showed no calcification or plaque lesions in the aorta, and the ascending aorta was running just below the sternum [Fig. 2]. Furthermore, preoperative coronary catheter angiography showed no stenosis in the coronary arteries. Considering the risk of wound healing and infection due to the large blisters in the median chest wall and skin erosions in the anterolateral third and fourth intercostal spaces, the right thoracotomy approach was preferred in relatively healthy skin using totally endoscopic concomitant aortic valve replacement and mitral valve repair. Polyurethane and silicon dressing foams were used to protect the skin at the site of contact with the bag valve mask, arterial pressure catheter, intravenous catheter, and tracheal intubation tube [Fig. 3]. The tracheal intubation tube and the TEE probe were coated with a large amount of xylocaine jelly. A left semi-supine position at 15° and three skin incision in the chest were made. The main skin incision was 7 cm in the fourth intercostal space outside the nipple; the second skin incision was 5 cm in the second intercostal space; and the camera port was in the fourth intercostal space and the mid-axillary line [Fig. 4a]. After cardiopulmonary bypass (CPB) was established from the common femoral artery (an 18-Fr femoral cannula [Edwards, Irvine, CA, USA]) and vein (a 22-Fr QuickDraw™ single-stage venous cannula [Edwards, Irvine, CA, USA]) using a Seldinger-guided and TEE-guided technique, we added a 22-Fr right-angle venous cannula in the superior vena cava (SVC). Aortic cross-clamping was performed above the pulmonary artery. Cardiac arrest was achieved via retrograde cardioplegic perfusion (snaring SVC and inferior vena cava, and incision of the right atrium). Mitral annuloplasty and approximation of leaflets P1 and P2 were performed using a 28 mm Memo3D ReChord™ (Corcym, Group, Milan, Italy). The position of the camera was changed from the camera port to the main incision site. Aortic valve replacement (AVR) was performed using size S Perceval™ aortic prostheses (Corcym, Group, Milan, Italy). The sizer and prosthesis were inserted from the second incision to maintain the coaxial axis [Fig. 4b–d].

Intraoperative TEE revealed adequate coaptation of the mitral valve, while mitral valve regurgitation and paravalvular leakage were absent. The skin incisions were closed with vertical mattress sutures using 2–0 nylon suture, and draping was carefully removed using a remover owing to skin fragility. The CPB, aorta cross-clamping, and operation times were 269 min, 188 min, and 416 min, respectively. Before extubation, the otolaryngologist confirmed with the fiber that no mucosal damage had occurred.

**Fig. 5:**
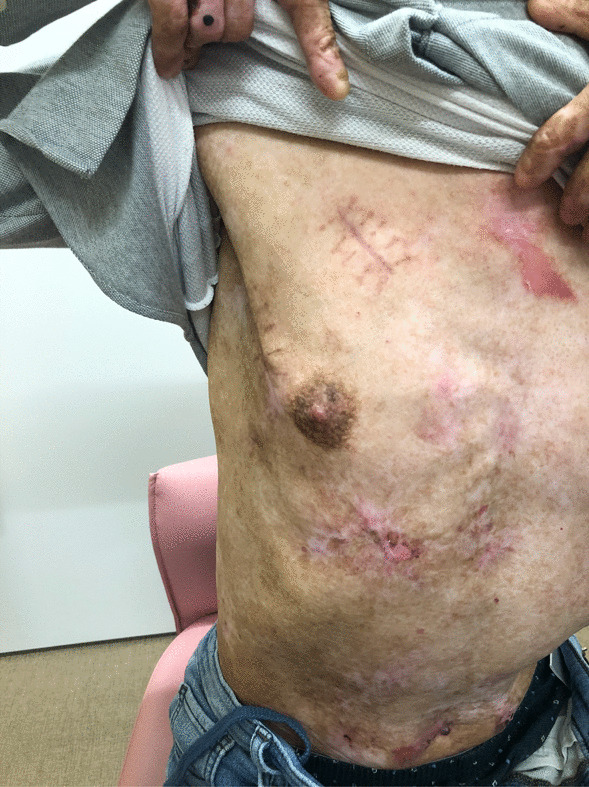
Healing wounds

The postoperative TTE showed that LVDd/Ds were 41/26 mm, paravalvular leakage (PVL) was absent, mean pressure gradient of Perceval was 19.7 mmHg, and residual mitral valve regurgitation was trivial. The surgical wounds healed [Fig. 5] and the patient was discharged nine days after the operation. The rest of the postoperative course was uneventful and reoperation and wound trouble was not seen after a 3-year follow-up period.

## Discussion and conclusion

EB is a group of hereditary skin diseases characterized by blistering of the skin, induced by mild trauma. Blistering results in open wounds that predispose to both scarring and infection [1, 3]. The four main types of EB are as follows: epidermolysis bullosa simplex, dystrophic epidermolysis bullosa, JEB, and Kindler syndrome [1]. JEB is the major form of EB, which is a generalized severe form of the condition, with a poor prognosis [1, 2]. Affected individuals experience blistering over large regions of the body from birth or early infancy. Blistering also affects mucous membranes, such as the moist lining of the mouth and digestive tract, which can cause difficulty in eating and digesting food.

The milder form of junctional epidermolysis bullosa is called the JEB generalized intermediate and is typically associated with a better prognosis [2]. Junctional epidermolysis bullosa most commonly results from mutations in the LAMA3, LAMB3, LAMC2, and COL17A1 genes. Mutations in each of these genes can cause JEB-generalized severe or JEB-generalized intermediates [2]. There is currently no specific treatment for EB. There are two reports in the literature on breast cancer treatment in patients with EB [4, 5]; however, there are no reports of cardiac surgery.

Because of skin and mucosal fragility, careful attention should be paid not only to the surgical technique, but also to anesthesia induction [5]. To protect the skin and mucosa, polyurethane and silicon dressing foams should be used at the contact sites, and a large amount of xylocaine jelly is better suited for use in tracheal intubation tubes and TEE probes. Surgical instruments that could potentially tear the skin, such as forceps, should be substituted for skin hooks or use of hands to perform suturing with vertical mattress sutures, and draping should be carefully removed using a remover. The surgical wounds had good healing using our strategy.

Both the aortic and mitral valves were treated. Median sternotomy for aortic and mitral valve surgery is performed classically, but the present case had skin fragility and a large blister in the median chest wall, which was at high risk for mediastinitis such as atopic dermatitis. Endoscopic surgical mitral valve repair is a difficult but established technique [6, 7]. Hence, the main skin incision was placed in the fourth intercostal space outside the nipple, the second skin incision was in the second intercostal space, and the camera port was in the fourth intercostal space and mid-axillary line, considering the skin condition and the running of the aorta. We did not choose the anterolateral large incision in the third or fourth intercostal space because of several erosions [Fig. 4] and decided to use the Perceval™ prosthesis for reliable insertion and implantation. After mitral valve repair, the camera position was changed from the port to the main incision site. These strategies improved the camera view of the aortic valve, and the sizer and prosthesis inserted from the second incision maintained the coaxial axis properly. Additionally, incisions in the skin that are in a relatively good condition contributed to healing without infection.

The American College of Cardiology/American Heart Association guidelines state that the choice of mechanical versus bioprosthetic valve replacement for patients aged between 50 and 65 years should be made in a shared decision-making process that must account for the trade-offs between durability (and the need for reintervention), bleeding, and thromboembolism [8]. The 2021 European Society of Cardiology and European Association for Cardio-Thoracic Surgery Guidelines state that a mechanical prosthesis should be considered in patients aged < 60 years for prostheses in the aortic position [9]. The patient was 55 years old; however, given the fragility of the skin and mucosa, the risk of bleeding due to lifelong anticoagulation therapy is considered higher than in other patients, so the use of a bioprosthetic valve was preferred after thorough informed consent from the patient.

The Perceval sutureless bioprosthesis is constructed from bovine pericardium fixed in a metal cage composed of an alloy of nickel and titanium. Szecel et al. reported that after more than 11 years of continued clinical use of the Perceval sutureless valve, they observed low mortality and stroke rates, and none for structural valve degeneration with good hemodynamic behavior of the valve [10]. A meta-analysis revealed no significant difference in early mortality, perioperative complications, and paravalvular leakage between the Perceval and conventional prosthesis groups. Moreover, a shorter CPB time, aortic cross-clamping time, and higher pacemaker implantation rate were observed in the Perceval group [11]. Some reports revealed that the authors did not experience more failures or PVL when using Perceval in multiple valve procedures, which corroborates recent findings [10, 12]. We decided to use the Perceval prosthesis because the insertion and implantation of the sutureless valve were easier than those of conventional prostheses in total endoscopic surgery. The sutureless technique is advantageous for preventing PVL in totally endoscopic minimally invasive cardiac surgery. In this patient, the postoperative mean pressure gradient of the Perceval prosthesis was higher than the previous reports [10]. There was no reoperation up to three years post-operation; however, close follow-up based on TTE is mandatory to detect prosthesis failure.

In conclusion, totally endoscopic minimally invasive concomitant aortic and mitral valve surgery using Perceval prosthesis can be performed safely for patients with junctional epidermolysis bullosa with adequate protection of the skin and mucosa.

## Data Availability

All data generated or analyzed are included in this article.

## Abbreviations

EB: Epidermolysis bullosa
JEB: Junctional epidermolysis bullosa
MICS: Minimal invasive cardiac surgery
TTE: Transthoracic echocardiography
LVDd: Left ventricular diastolic diameter
LVDs: Left ventricular systolic diameter
TEE: Transesophageal echocardiography
CPB: Cardiopulmonary bypass
SVC: Superior vena cava
PVL: Paravalvular leakage
AVR: Aortic valve replacement
